# Tumor protein D52 is upregulated in oral squamous carcinoma cells under hypoxia in a hypoxia-inducible-factor-independent manner and is involved in cell death resistance

**DOI:** 10.1186/s13578-021-00634-0

**Published:** 2021-07-03

**Authors:** Yuzo Abe, Yoshiki Mukudai, Mai Kurihara, Asami Houri, Junichiro Chikuda, Atsutoshi Yaso, Kosuke Kato, Toshikazu Shimane, Tatsuo Shirota

**Affiliations:** Department of Oral and Maxillofacial Surgery, School of Dentistry, Showa University, 2-1-1 Kitasenzoku, Ota-ku, Tokyo, 145-8515 Japan

**Keywords:** TPD52, Hypoxia, HIF, Squamous cell carcinoma, RNA stability, Autophagy

## Abstract

**Background:**

Tumor protein D52 (TPD52) reportedly plays an important role in the proliferation and metastasis of various cancer cells, including oral squamous cell carcinoma (OSCC) cells, and is expressed strongly at the center of the tumor, where the microenvironment is hypoxic. Thus, the present study investigated the roles of TPD52 in the survival and death of OSCC cells under hypoxia, and the relationship with hypoxia-inducible factor (HIF). We examined the expression of TPD52 in OSCC cells under hypoxic conditions and analyzed the effects of HIF on the modulation of TPD52 expression. Finally, the combinational effects of TPD52 knockdown and HIF inhibition were investigated both in vitro and in vivo.

**Results:**

The mRNA and protein levels of TPD52 increased in OSCC cells under hypoxia. However, the increase was independent of HIF transcription. Importantly, the observation was due to upregulation of mRNA stability by binding of mRNA to T-cell intercellular antigen (TIA) 1 and TIA-related protein (TIAR). Simultaneous knockdown of TPD52 and inhibition of HIF significantly reduced cell viability. In addition, the in vivo tumor-xenograft experiments showed that TPD52 acts as an autophagy inhibitor caused by a decrease in p62.

**Conclusions:**

This study showed that the expression of TPD52 increases in OSCC cells under hypoxia in a HIF-independent manner and plays an important role in the proliferation and survival of the cells in concordance with HIF, suggesting that novel cancer therapeutics might be led by TPD52 suppression.

**Supplementary Information:**

The online version contains supplementary material available at 10.1186/s13578-021-00634-0.

## Background

The tumor protein D52 (TPD52) family of proteins includes TPD52 [1, 2], TPD53 [1, 3–5], TPD54 [4, 5], and TPD55 [6]. TPD52 was identified more than 25 years ago [7] and revealed through overexpression of its coding gene in breast and lung cancer [7, 8]. Other family members, such as TPD53 (also known as TPD52L1), TPD54 (TPD52L2), and TPD55 (TPD52L3), have been reported to be highly expressed in ovary [9–11], testis [12, 14], colon [15, 16], and prostate cancer [2, 15], as well as in brain tumors [16], lymphoma [17], and leukemias [17, 18]. We reported that TPD54 is a negative regulator of extracellular-matrix-dependent migration and cell attachment in oral squamous carcinoma cells [19]. Among the family members, TPD52 has been studied the most, due to its role in the malignancy of various cancer cells. A previous study reported that overexpression of TPD52 induces proliferation of non-malignant 3T3 fibroblasts and promotes scaffold-independent cell proliferation [20]. In addition, overexpression of TPD52 was reported to increase tumor growth, demonstrating its physiological and pathological role. Shang et al*.* [21] showed that the expression of PC-1/PrLZ, a splicing variant of human TPD52, is increased by stress from irradiation. Moreover, we revealed that TPD52 is strongly expressed at the center of OSCC tissue and plays an important role in OSCC cell growth [19]. Another report of ours represented that TPD52 is post-transcriptionally regulated by T-cell intercellular antigen (TIA) 1 and TIA-related protein (TIAR) via mRNA stability [22]. However, the detailed roles of TPD52 in the proliferation and survival of cancer cells are still unclear.

Solid tumors have microenvironments that are exposed to hypoxia (reviewed in [23]). Cancer cells are difficult to treat as they are highly resistant to radiation, drug therapies, and hypoxia. Hypoxia-inducible factor (HIF)-1 is a heterodimer composed of α and β subunits, and was identified as a transcription factor that promotes the production of erythropoietin [24]. HIF-1α is hydroxylated by prolyl hydroxylase domain proteins (PHDs) in normoxia, and hydroxylated HIF-1α is ubiquitinated by von Hippel-Lindau protein (pVHL), followed by proteasomal degradation. HIF-1α also undergoes asparagine hydroxylation by factor inhibiting HIF-1 (FIH), whereby HIF-1α inhibits binding to p300/CBP. Under hypoxia, the activity of PHDs and FIH is strongly restrained, and HIF-1α translocates into the nucleus and forms a heterodimer with HIF-1β. Angiogenesis is induced by binding the heterodimer to the hypoxic response sequence (HRE) of the target gene (reviewed in [25]). In addition, HIF-1 activation is known to be involved in infiltration, metastasis, metabolic reprogramming, resistance to chemotherapy, and radiation therapy [26].

Recently, Wang et al. [27] reported a hypoxia-related prognostic signature for breast cancer that included overexpression of TPD52. In line with these results, we planned the present study to investigate whether TPD52 plays an important role in the growth and survival of OSCC cells under hypoxia, a common microenvironment feature in cancer. We also investigated the relationship between TPD52 and HIF. As a result, it was found that upregulation of TPD52 was independent of HIF and was regulated post-transcriptionally by binding to TIA-1 and TIAR. Further, we found that knockdown of TPD52 and inhibition of HIF synergistically suppressed the growth and induced the cell death of OSCC cells both in vitro and in vivo.

## Results

### TPD5 2 is induced under hypoxia

**Fig. 1:**
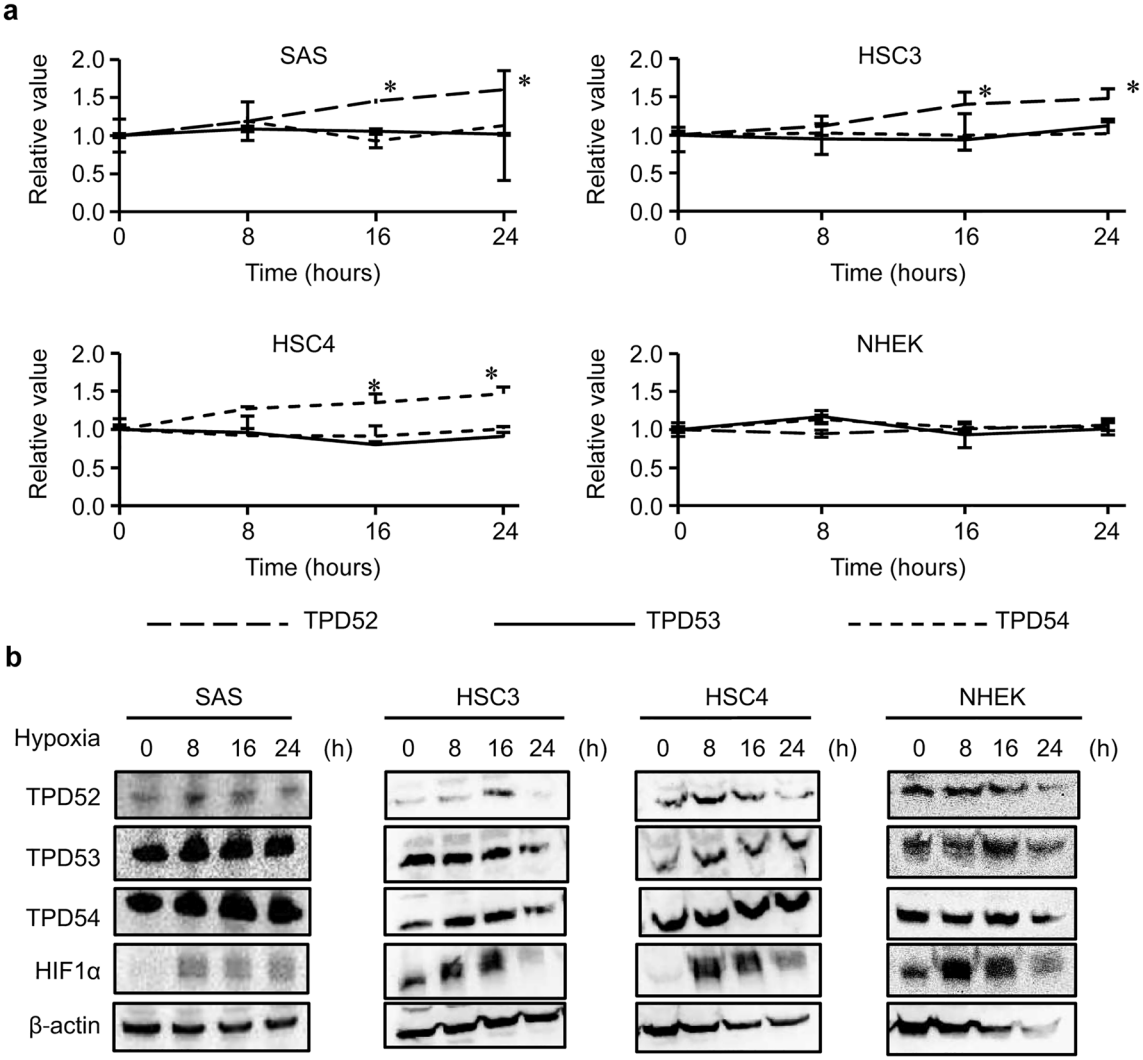
Induction of TPD52, 53, and 54 in OSCC cells and NHEK cells using hypoxia. SAS, HSC3, HSC4, and NHEK cells were exposed to hypoxia for 0, 8, 16, and 24 h. Then, TPD52, TPD53, and TPD54 levels were analyzed using RT-qPCR (**a**) and TPD52, TPD53, TPD54, HIF-1α, and β-actin levels were analyzed using western blotting (**b**). For RT-qPCR, the value at time 0 is designated as “1,” and relative values are shown. *, *p* < 0.05 versus time 0. Experiments were repeated 3 times

Our recent study [19] showed strong expression of TPD52 at the center of the tumor, and Additional file 3: Fig. S1 shows that in the specimen of tongue OSCC tumor, TPD52, 53 and 54 was expressed at the almost same region, where HIF-1α, a marker of hypoxia, was expressed. Thus, we examined whether hypoxia induces the expression of TPD52 family genes and proteins in OSCC cells and normal keratinocytes (Fig. 1). Only *TPD52* mRNA was increased by hypoxia in all OSCC cells (SAS, HSC3, and HSC4) in a time-dependent manner, while TPD53 and 54 mRNAs were barely increased (Fig. 1a). However, in NHEK cells, no mRNAs were increased by hypoxia. Western blotting (Fig. 1b) showed similar results regarding protein expression. In SAS cells, TPD52 mRNA and protein levels increased the most prominently. Thus, we used SAS cells as a representative of OSCC cells in the following experiments. These results suggest that TPD52 may play an important role in OSCC cells, not in normal keratinocytes, under hypoxia, compared to TPD53 and 54.

### TPD52 increase by hypoxia is independent of HIF and transcription

**Fig. 2:**
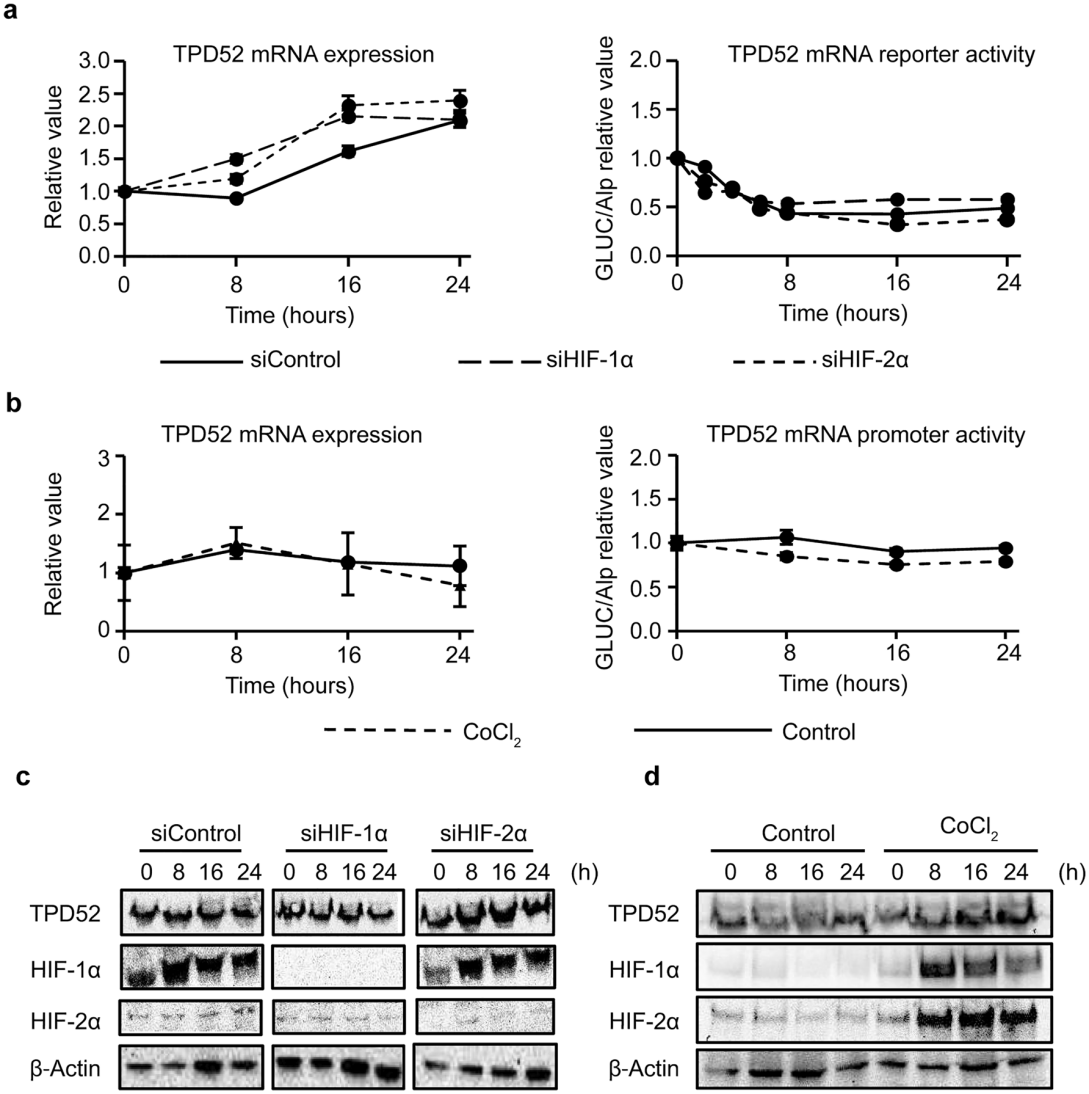
Effect of knockdown of HIF-1/2α and induction of HIF. **a** siRNAs for HIF-1α or 2α, or control siRNA were transfected into SAS cells, and incubated under normoxia conditions for 24 h. Then, the cells were exposed to hypoxia for 0, 8, 16, and 24 h, and were subjected to RT-qPCR and reporter assay to determine TPD52. The value at time 0 is designated as “1,” and relative values are shown. **b** SAS cells were exposed to normoxia for 0, 8, 16, and 24 h, in the presence or absence of 10 μM CoCl_2_. Then, the cells were subjected to RT-qPCR and reporter assays to detect TPD52. The value at time 0 is designated as “1,” and relative values are shown. **c** siRNAs for HIF-1α or 2α, or control siRNA were transfected into SAS cells, and the cells were exposed to hypoxia for 0, 8, 16, and 24 h. Then, the cells were subjected to western blotting analysis to detect TPD52, HIF-1α, HIF-2α, and β-actin. **d** SAS cells were exposed to normoxia for 0, 8, 16, and 24 h, in the presence or absence of 10 μM CoCl_2_. Then, the cells were subjected to western blotting analysis to detect TPD52, HIF-1α, HIF-2α, and β-actin. Experiments were repeated 3 times

When various cells are exposed to hypoxia, HIF-1α is upregulated and gene transcription is activated downstream of HIF (reviewed in [31]). Thus, we hypothesized that the increase of TPD52 under hypoxia is regulated by HIF, and we examined the effects of HIF-1/2α knockdown on the expression and promoter activity of TPD52 under hypoxia (Fig. 2a). Surprisingly, *TPD52* mRNA increased under hypoxia despite HIF-1/2α knockdown, as shown in RT-qPCR. Moreover, the reporter assay showed that the promoter activity of TPD52 appeared decreased by hypoxia, but also that this observation was not modulated by HIF-1/2α knockdown. The results indicate that an increase in *TPD52* mRNA is independent of the HIF pathway and the transcriptional level. In order to reinforce the independence of *TPD52* mRNA from HIF, the cells were incubated under normoxia in the presence of CoCl_2_, a HIF activator, and then the same assays were performed (Fig. 2b). The addition of CoCl_2_ to normoxia did not increase the mRNA or transcriptional activity of TPD52, indicating that the expression of TPD52 is not located downstream of HIF. Western blotting analysis (Fig. 2c and d) showed successful knockdown by siRNA and successful activation of HIF-1/2α by CoCl_2_.

### Increase of *TPD52* mRNA under hypoxia is regulated by the upregulation of mRNA stability via binding to TIA-1 and TIAR

**Fig. 3:**
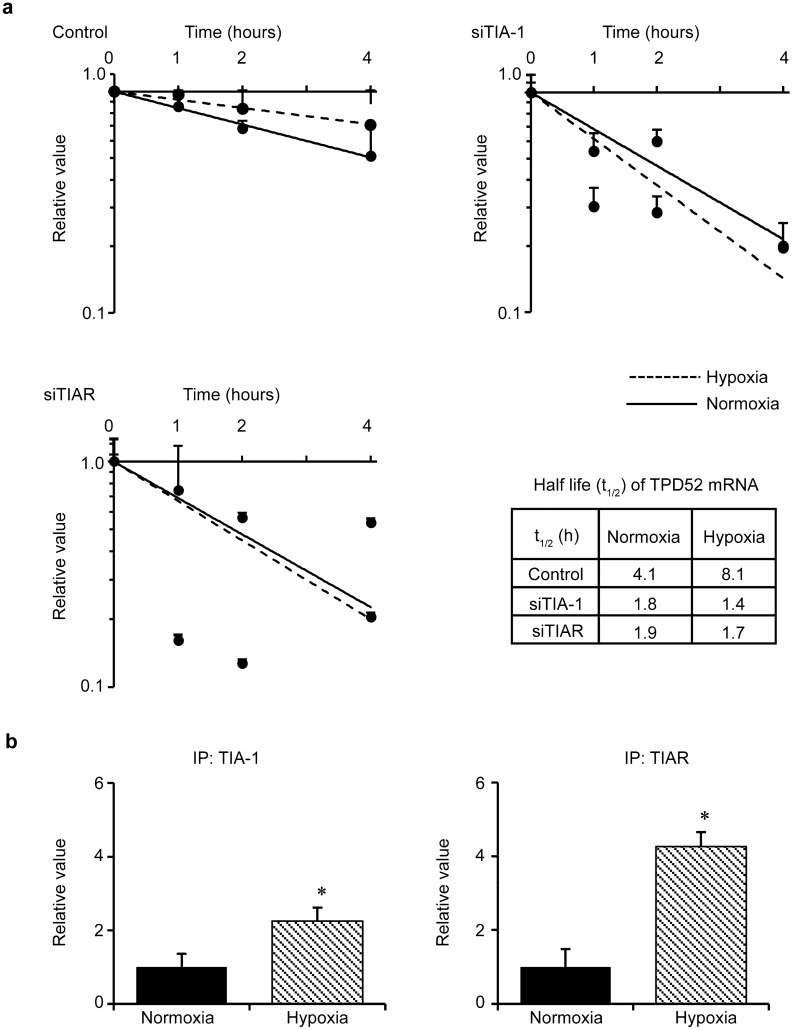
Effects of knockdown of TIA-1 and TIAR on stability of *TPD52* mRNA. **a** RNA degradation assay. siRNAs for TIA-1 and TIAR, and control siRNA were transfected into SAS cells. After the addition of actinomycin D (10 μg/ml) to the culture, the cells were incubated under hypoxic conditions for another 0, 1, 2, and 4 h. Then, total RNA was collected, and RT-qPCR was performed to detect *TPD52* mRNA. The value at time 0 is designated as “1,” and relative values are shown. Bottom right: calculated half-lives (t_1/2_) of *TPD52* mRNA are shown. **b** RIP assay. SAS cells were exposed to normoxia or hypoxia for 24 h. Then, the cells were subjected to RIP assays to detect *TPD52* mRNA, using anti-TIA-1 or anti-TIAR antibodies. The value of normoxia is designated as “1,” and relative values are shown. *, *p* < 0.05. Experiments were repeated 3 times

Since our previous study [22] demonstrated that TPD52 is post-transcriptionally regulated by TIA-1 and TIAR via mRNA stability, we examined the stability of *TPD52* mRNA under hypoxic conditions using the RNA degradation assay (Fig. 3a). The t_1/2_ of *TPD52* mRNA under normoxia was 4.1 h, while that under hypoxia extended to 8.1 h (in 2 folds). Under normoxia, knockdown of TIA-1 and TIAR decreased the stability of *TPD52* mRNA by approximately ½-fold. Interestingly, the increased stability of *TPD52* mRNA by hypoxia was drastically (more than 4 folds) abolished by knockdown of TIA-1 and TIAR (8.1 h, (control) versus 1.4 (siTIA-1) and 1.7 h (siTIAR)). The RIP assay (Fig. 3b) showed that hypoxia reduced the binding abilities of TIA-1 and TIAR to *TPD52* mRNA, suggesting that modulation of binding ability may regulate the stability of *TPD52* mRNA. Additional file 3: Fig. S1 shows that TIA-1 and TIAR aggregated and formed stress granules (SGs) under hypoxia. Thus, the aggregation of TIA-1 and TIAR may result in decreased binding ability to *TPD52* mRNA by forming SGs.

### TPD52 plays an important role in the proliferation and apoptosis of OSCC cells under hypoxia

**Fig. 4:**
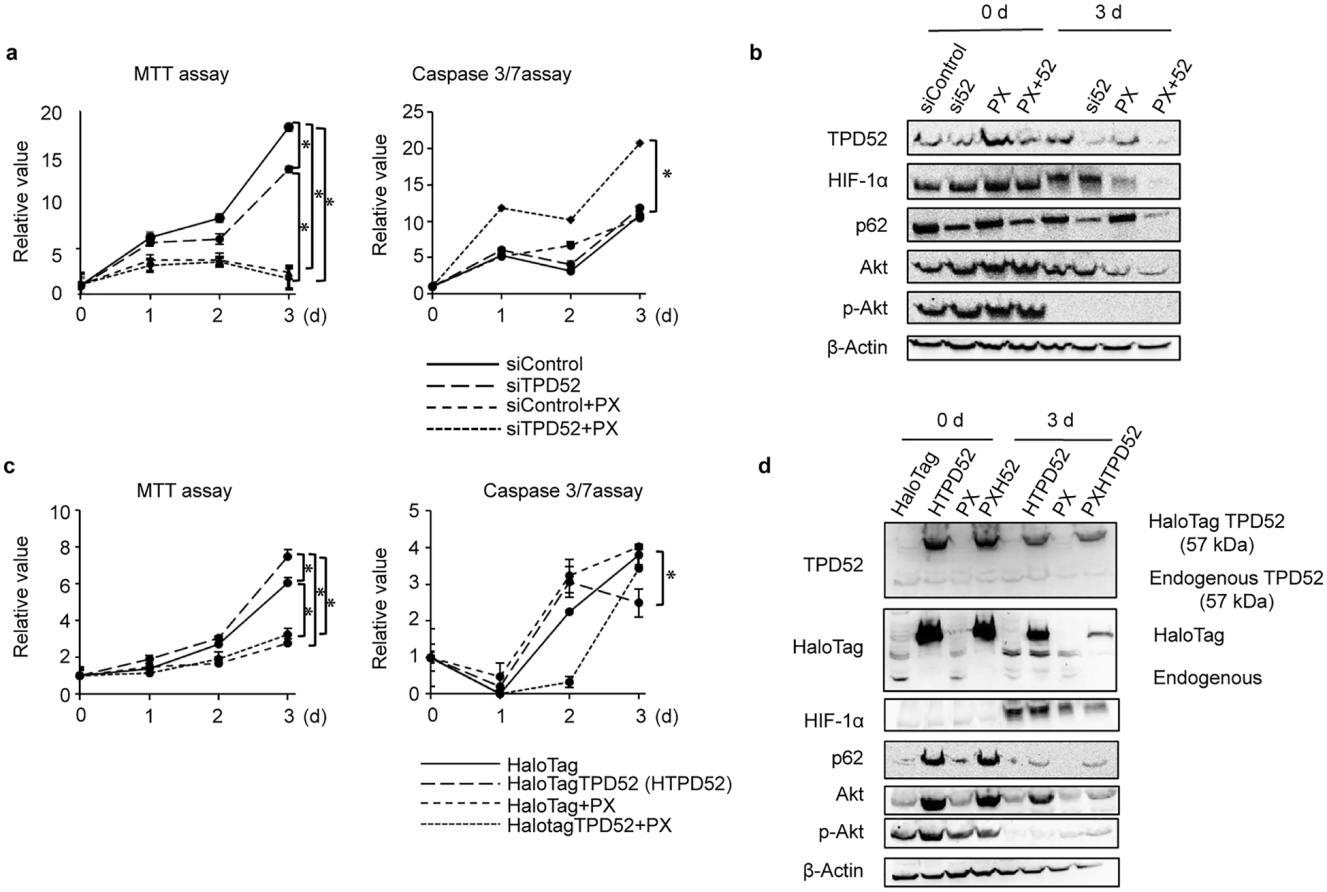
Effects of knockdown and overexpression of TPD52 on growth and apoptosis of SAS cells. **a**, **b** siRNA for TPD52 or control siRNA was transfected into SAS cells, and incubated under normoxic conditions for 24 h. Then, the cells were exposed to hypoxia in the presence or absence of 10 μM PX-478. After 0, 1, 2, and 3 d cells were subjected to MTT and caspase 3/7 assays (**a**), and after 0 and 3 d, total cellular proteins were subjected to western blotting analysis to determine expression of TPD52, HIF-1α, p62, Akt, p-Akt, and β-actin (**b**). **c**, **d** HaloTag-TPD52 or control HaloTag vector was transfected into SAS cells, and incubated under normoxic conditions for 24 h. Then, the cells were exposed to hypoxia in the presence or absence of 10 μM PX-478. After 0, 1, 2, and 3 d, cells were subjected to MTT and caspase 3/7 assays (c), and after 0 and 3 d, total cellular proteins were subjected to western blotting analysis to detect TPD52 (overexpressed and endogenous proteins indicated with arrows with the molecular weight), HIF-1α, p62, Akt, p-Akt, HaloTag, and β-actin (**d**). For MTT and caspase 3/7 assays, the value at time 0 is designated as “1,” and relative values are shown. The values of 3 d were subjected to ANOVA. *, *p* < 0.05. Experiments were repeated 3 times

The role of increased TPD52 under hypoxia was investigated using MTT and caspase 3/7 assays. TPD52 knockdown (Fig. 4a) decreased MTT activity, indicating decreased cell proliferation and/or cell viability. In contrast, increased caspase 3/7 activity indicated increased apoptosis. The addition of PX-478, a HIF-inhibitor, to the culture showed synergistic effects regarding decreasing MTT activity and increasing caspase 3/7 activity. Next, the cells were subjected to overexpression of TPD52 (Fig. 4c). Overexpression of TPD52 resulted in increased MTT activity and decreased caspase 3/7 activity, reflecting the opposite effects of knockdown. Additionally, PX-478 decreased MTT activity and increased caspase 3/7 activity, regardless of TPD52 overexpression. Western blotting analysis (Fig. 4b and d) shows successful knockdown and overexpression of TPD52 by transfection and inhibition of HIF by the addition of PX-478. These results strongly suggest an important role for the survivability of OSCC cells under hypoxia.

### Knockdown of TPD52 and inhibition of HIF-1α synergistically suppresses tumor growth

**Fig. 5:**
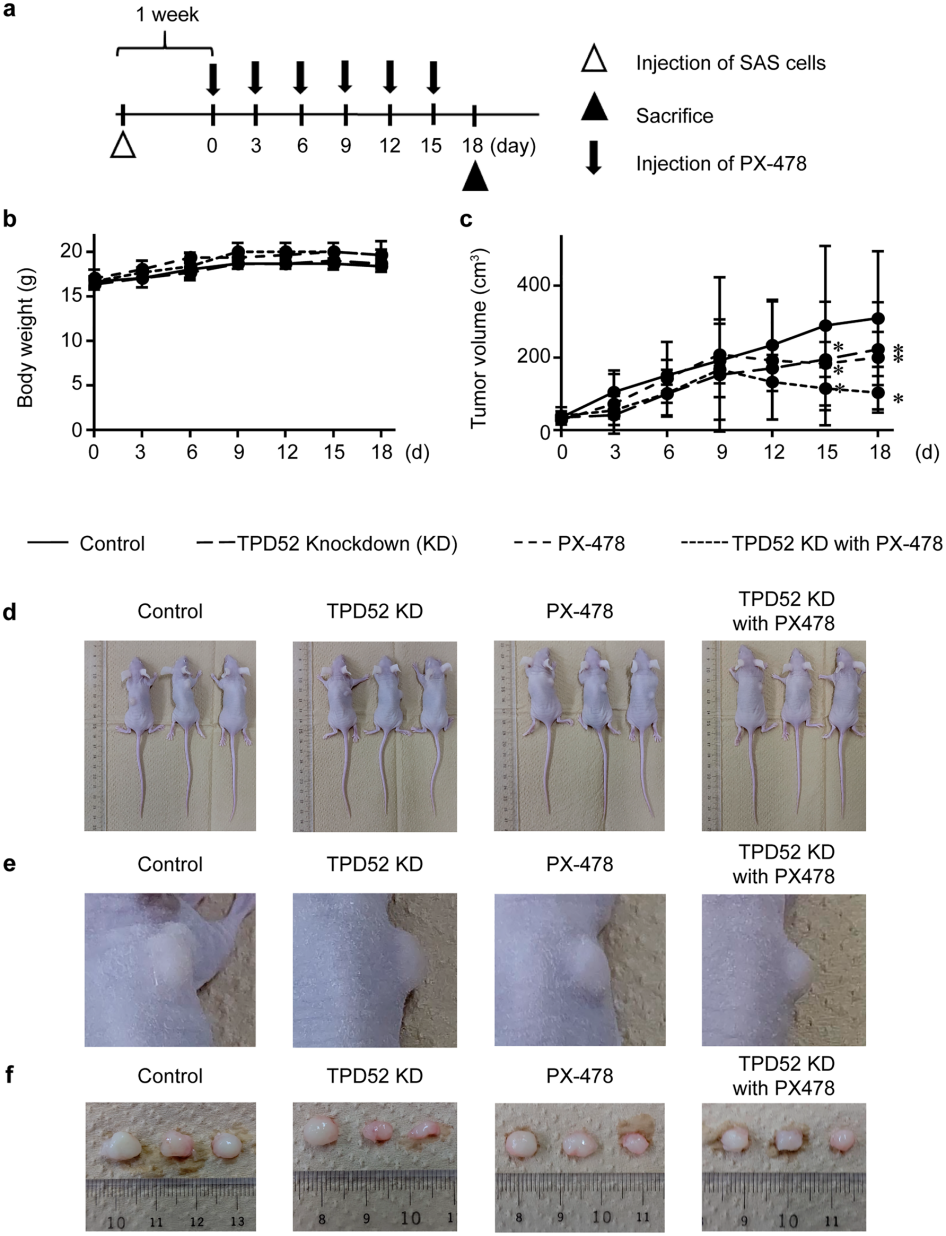
Synergistic effects of TPD52 knockdown and inhibition of HIF in tumor-xenograft mouse models. **a** The experimental schema. TPD52-knockdown or control SAS cells were xenografted in a unilateral flank of mice (open triangle). After 1 w of maintenance, this day was designated as “0 d.” Saline or 0.5 μg/kg bodyweight of PX-478 were injected intraperitoneally, and bodyweight and tumor volumes were measured every three days (arrow). On day 18, the mice were euthanized, and the tumor was collected (closed triangle). **b** Average body weight changes observed in the mice. **c** Average tumor volume changes in the mice. *, *p* < 0.05 *versus* control. **d**, **e** and **f** Photographic images of tumor-xenografted mice and collected tumors on day 18. Experiments were repeated 3 times

**Fig. 6:**
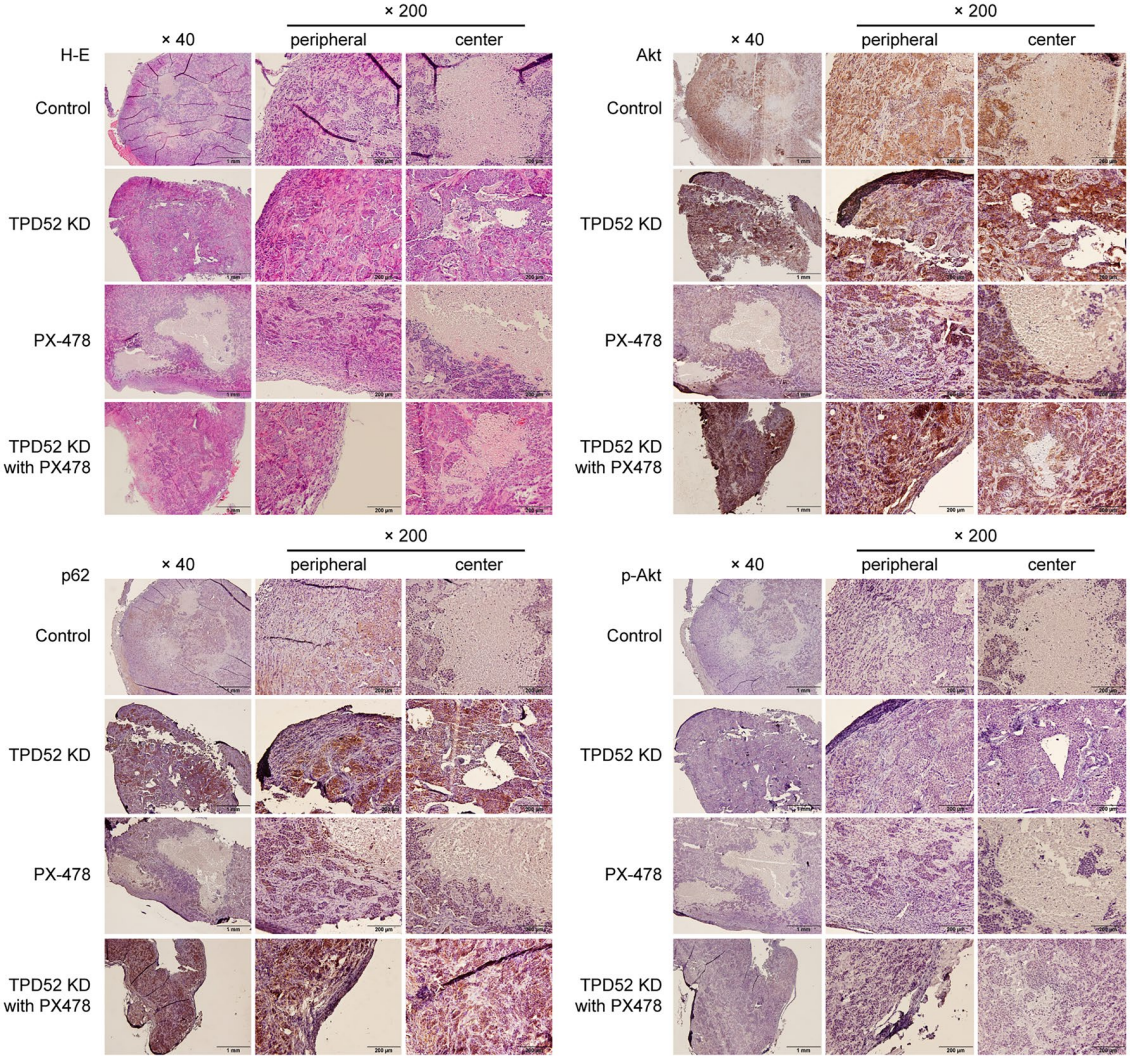
Histopathological images of the tumors from tumor-xenografted mice. Collected tumors (Control, TPD52KD, PX-478, and TPD52KD with PX-478) were subjected to HE staining and immunohistochemistry to detect p62, Akt, and p-Akt. Images of the peripheral and center of the tumor are shown individually. Bars, 1 mm (× 40) and 200 mm (× 200). Experiments were repeated 3 times

Finally, we examined the synergy of TPD52 knockdown and inhibition of HIF in vivo by employing tumor xenograft mice (Fig. 5). Neither knockdown of TPD52 nor inhibition of HIF showed significant differences in body weight or survival of mice until day 18 (Fig. 5b). However, knockdown of TPD52 reduced tumor volume, and inhibition of HIF showed a stronger effect. Notably, knockdown of TPD52 in addition to inhibition of HIF drastically decreased tumor volume, indicating a strong synergistic effect (Fig. 5c–f). Next, the histopathology of the tumor on day 18 was examined (Fig. 6). HE staining showed that tumor centers in the TPD52 and PX-478 knockdown groups were sparse, suggesting necrotic cell death by hypoxia. Furthermore, this observation was the most remarkable in the TPD52 knockdown group with PX-478, indicating a synergistic effect on necrosis. Immunohistochemical analysis showed that p62 was expressed at the periphery of the tumor, even in the control group, suggesting that autophagy may lead the cells to resist cell death. In the knockdown of TPD52 and PX-478 groups, the expression of p62 was stronger, and this was the most prominent in knockdown of TPD52 with PX-478. Total Akt was also the most strongly expressed in the group with TPD52 knockdown and PX-478, although p-Akt was faint in all groups. Taken together, these results indicate that knockdown of TPD52 reduced tumor cell viability at the hypoxic center of the tumor, and that PX-478 showed similar effects. However, the synergistic effect of TPD52 knockdown and inhibition of HIF drastically reduced tumor cell viability at the center of the tumor. In addition, it is suggested that knockdown of TPD52 may induce autophagy and necrotic cell death resistance.

## Discussion

Several functions of TPD52 have been studied in various cancer cells [1–21]. In addition, overexpression of TPD52 was reported by systematic analysis of a hypoxia-related prognostic signature for breast cancer [27]. However, the role of TPD52 in hypoxia has never been reported, to the best of our knowledge. The central tumor region is exposed to hypoxia, whereby the cancer cells obtain the ability to resist radiation and drug therapies [32]. Our previous study reported that TPD52 is strongly expressed at the center of OSCC. Similarly, a histological specimen of a tongue OSCC tissue (Additional file 3: Fig. S1) shows the expression of TPD52 at a hypoxic region in the tumor. Then, we examined whether hypoxic conditions induce the expression of the TPD family (Fig. 1). Only TPD52 was increased by hypoxia in OSCC cells at both the mRNA and protein levels. The decrease in TPD52 protein levels at 24 h might be due to the beginning of death of OSCC cells caused by a hypoxic environment. However, this was not observed in NHEK cells. Moreover, TPD52 mRNA and protein levels increased most prominently in SAS cells. These results suggest that TPD52 may be involved in the survival and proliferation of OSCC cells.

HIF is known to be an important factor for the survival of various cancer cells under hypoxic conditions [32] and is involved in infiltration (reviewed in [33]), metastasis [34], metabolic reprogramming, and resistance to chemo and radiation therapies [26]. Further, anticancer drugs targeting HIF are currently being developed (reviewed in [35]). We investigated whether increased expression of TPD52 under hypoxia was regulated by HIF (Fig. 2). Contrary to our expectation, knockdown of HIF-1/2α did not increase TPD52 mRNA and protein under hypoxia. In addition, the promoter assay for TPD52 showed not only that TPD52 promoter activity was decreased by hypoxia, but also that knockdown of HIF-1/2α showed little effect. These results indicate that the increase in TPD52 under hypoxia is independent of transcription. HIF-1α is always degraded by the action of PHDs under normoxia, and the activity of PHDs is decreased by hypoxia due to lack of O_2_ [36]. Cobalt is a well-known PHD inhibitor, which induces the expression of HIF-1α via the phosphoinositide 3-kinase (PI3K) pathway, resulting in the activation of HIF-1α even under normoxia [37]. Therefore, we carried out the next experiment, where HIF-1α was induced by the addition of CoCl_2_ under normoxia. However, this did not increase *TPD52* mRNA, nor did it increase TPD52 promoter activity. These results reinforced the observation that TPD52 is HIF-independent and transcription-independent.

We also reported [22] that the expression of *TPD52* mRNA is post-transcriptionally regulated through the stability of TIA-1 and TIAR, major components of SGs [38]. Stress granules are structures that are temporarily formed in the cytoplasm by stress stimuli, such as hypoxia [39], endoplasmic reticulum stress [40], heat shock [41], and viral infection [40]. We examined whether the increase in *TPD52* mRNA resulted from an increase in mRNA stability, and, if so, whether TIA-1 and TIAR were involved. Therefore, we carried out RNA degradation and RIP assays. As a result, hypoxia increased the stability of *TPD52* mRNA by approximately twofold. The downregulation of TIA-1 and TIAR decreased stability under normoxia, in agreement with our previous study [22]. Interestingly, under hypoxia, the knockdown of these genes showed drastic effects, whereby mRNA increased by hypoxia stability was abolished more than that observed in normoxia. The RIP assay showed that hypoxia reduced the binding abilities of TIA-1 and TIAR to *TPD52* mRNA. Therefore, it was suggested that the increase in *TPD52* mRNA under hypoxia may be due to increased binding of TIA-1/TIAR, which was triggered by hypoxic stress. TIA-1 and TIAR are important components of SGs [38]. Translation efficiency, mRNA turnover, and mRNA stability are regulated by SG formation [42]. Additional file 3: Fig. S2 shows the aggregation of TIA-1 and TIAR in the cytosol of the cells under hypoxia, indicating the formation of SGs. Thus, the involvement of SG formation in the increase of *TPD52* mRNA might be shown in the present study, although further investigation is required.

In order to investigate the proliferation, survival, and apoptosis of TPD52 on OSCC cells exposed to hypoxic conditions, we investigated the combined effects of TPD52 knockdown and inhibition of HIF (Fig. 4). PX-478, a chemical inhibitor of HIF, inhibits the translation of HIF-1α [43]. TPD52 knockdown or PX-478 alone led to reduced MTT activity and increased caspase 3/7 activity under hypoxia. However, the combined use of these drastically increased the effects. Since the repressing effects of TPD52 knockdown and inhibition of HIF are thought to be independent of each other, as shown in the previous subsection, the result may be due to the interception of two (or possibly more) pathways. Next, the combined effects of TPD52 overexpression and the addition of PX-478 were examined. Overexpression of TPD52 alone increased MTT activity and decreased caspase 3/7 activity, showing the opposite effects of knockdown. This result showed that expression of TPD52 increased survivability of OSCC cells. The detailed function is still unclear and further investigation is needed. However, the effects of TPD52 overexpression were almost entirely abolished. This may be triggered by HIF inhibition, thereby resulting in the loss of cell viability maintenance. Therefore, we hypothesized that the combined effects can reduce xenografted tumor and moved on to in vivo studies.

TPD52 knockdown with the addition of PX-478 in tumor-xenograft mice demonstrated decreased tumor volume, as observed in the in vitro study (Fig. 5). These results suggest the possibility of a clinical application for cancer therapeutics. In immunohistochemistry, knockdown of TPD52 and addition of PX-478 each induced cell death at the center of the tumor, where the environment was most hypoxic. This also increased the expression of p62 and Akt at the tumor periphery. p62 is an autophagy-related protein that is reported to accumulate in the cytosol during autophagy failure [44]. Akt is related to macro-autophagy, and the activation of Akt initiates autophagy, followed by the consumption of p62 and formation of autophagosomes [45]. Conversely, Shang et al*.* reported [21] that suppression of TPD52 induces autophagy-induced cell death during irradiation, resulting from the consumption of p62, while Zhao et al*.* [46] reported that knockdown of TPD52 decreased Akt. Therefore, the results of the present study suggest that TPD52 may inhibit autophagy signaling by modulating the Akt signaling pathway. However, inhibition of HIF with knockdown of TPD52 might induce cell death in cancer cells under hypoxia due to cellular starvation through aberrant acceleration of autophagy.

HIF is reportedly involved in the growing malignancy of various cancer cells through upregulation of survivability under hypoxia [33] and is thought to be a molecular target for cancer therapeutics [36]. However, several groups, including ours, have studied the role of TPD52 in the proliferation and survival of various cancer cells [1–19].

## Conclusions

In the present study, we focused on the roles of TPD52 in OSCC cells under hypoxia. As a result, we first revealed that TPD52 is increased under hypoxia in a HIF-independent manner, and that the combination of TPD52 knockdown and HIF inhibition reduced cell viability and induced cell death, including apoptosis. These results may lead to novel cancer therapeutics by controlling the expression of TPD52 in cancer tissue. However, details regarding TPD52 in cancer cells under hypoxia are still to be investigated, and the following studies are now ongoing.

## Methods

### Cell culture

SAS [28], HSC 3 [29], and HSC 4 cells [29] (human oral squamous cell carcinoma-derived cell lines, kindly gifted by Dr. Ochiya, National Cancer Center) were grown in high glucose Dulbecco's modified Eagle's medium (HDMEM) with L-Glutamine and Phenol Red (Wako, Osaka, Japan), supplemented with 10% fetal bovine serum (FBS), 100 U/mL penicillin, and 100 mg/mL streptomycin at 37 °C, with 5% CO_2_ and 100% humidity. Normal human epidermal keratinocytes (NHEKs) were purchased from Promo Cell (Heidelberg, Germany) and grown in endothelial cell growth medium (Promo Cell) according to the manufacturer’s protocol. Hypoxic conditions were set at 37 °C, 2% O_2_ [30], and 5% CO_2_ in a BIOLABO mini-multi-gas-incubator (BL-43MD, TOSC, Tokyo, Japan).

### Antibodies

Rabbit monoclonal anti-TPD52 (ab182578) and anti-HIF-2α (ab199) antibodies were purchased from Abcam (Branford, CT, USA). Rabbit polyclonal anti-TPD53 (14732-1-AP), anti-TPD54 (11795-1-AP), anti-β-actin (20536-1-AP), and mouse monoclonal anti-TIAR (66907-1-Ig) antibodies were purchased from Proteintech (Rosemont, IL, USA). Rabbit monoclonal anti-HIF-1α antibody (#36169), anti-SQSTM1/p62 (#39749), anti-Akt1 (#2938), and anti-phospho-Akt (S473, #4060) were purchased from Cell Signaling (Danvers, MA, USA).

Rabbit polyclonal anti-TIA-1 antibody (RN014P) was purchased from MBL (Aichi, Japan). Rabbit polyclonal anti-HaloTag antibody (G9281) was purchased from Promega (Madison, WI, USA).

### Purification of cDNA synthesis and RT-qPCR

Total cellular RNA was purified using TRIzol Reagent (Life Technologies, Carlsbad, CA, USA) according to the manufacturer's protocol and stored at −30 °C until use. Total cellular RNA (100 ng) was reverse-transcribed using the ReverTra Ace qPCR RT Kit (TOYOBO, Osaka, JAPAN) according to the manufacturer's protocol. The generated cDNA was subjected to RT-qPCR using the KAPA SYBR FAST qPCR Kit (Kapa Biosystems, Boston, MA, USA), according to the manufacturer's protocol. For qPCR, statistical analysis was performed using the CFX Connect Real-Time System (BioRad, Hercules, CA, USA). The fold change in gene expression was calculated using the 2^−∆∆Ct^ method. Gene expression was normalized to 18 s rRNA for the RNA degradation assay or β-actin for other assays within each sample group. All primer sequences are shown in Additional file 2: Table S1.

### Protein preparation and western blot analysis

Total cellular proteins were prepared as previously described [19]. For western blot analysis, 20 mg of cellular protein was subjected to sodium dodecyl sulphate–polyacrylamide gel electrophoresis (SDS-PAGE) on a 4–15% gradient gel (Bio-Rad). The blot was transferred onto a polyvinylidene difluoride membrane using the iBlot 2 (Life Technologies), followed by blocking with Tris-buffered saline (Takara Bio, Shiga, Japan) containing 0.2% dry fat-free milk (Cell Signaling). Primary antibody reaction, horseradish-peroxidase-conjugated secondary antibody (NA934V, GE Healthcare UK Ltd., Buckinghamshire, UK) reaction, and washing steps have been previously described [30]. Bands were visualized using Amersham ECL western blotting detection reagent (GE Healthcare UK Ltd) and the ChemiDoc XRS Plus ImageLab system (Bio-Rad).

### Gene transfection

Small interfering RNAs (siRNAs) for human HIF-1α (EHU181981), HIF-2α (EHU008751), TPD52 (EHU130201), TIA-1 (EHU158111), TIAR (EHU069831), and control siRNA (for firefly luciferase, EHUFLUC) were purchased from Sigma–Aldrich (St Louis, MO, USA). The expression vectors of HaloTag-TPD52 (pFN21AE3730) and HaloTag control vector (G659) were purchased from Promega. The siRNAs and expression vectors were transfected with Lipofectamine 2000 (Life Technologies) according to the manufacturer's protocol.

### Reporter assay

A reporter vector (HPRM13144-PG04) of secreted *gaussia* luciferase (GLuc) downstream of the human TPD52 promoter region with secreted alkaline phosphatase (SEAP) downstream of the CMV promoter was purchased from GeneCopoeia (Rockville, MD, USA) and transfected into SAS cells with or without siRNAs as described above. The activities of GLuc and SEAP were measured using the Secrete-Pair Dual Luminescence Assay kit (GeneCopoeia) and GloMax-Multi + Detection System (Promega) according to the manufacturer’s protocol.

### RNA degradation assay

The assay was performed as described previously [22] by adding 10 mg/mL actinomycin D (Sigma-Aldrich) to the cell culture, as mentioned above, whole cell RNA was isolated at regular intervals (0, 1, 2, and 4 h) and used for RT-qPCR.

### RNA immunoprecipitation assays

The RNA immunoprecipitation (RIP) assay was performed using the RiboCluster Profiler RIP assay kit (MBL), anti-TIA-1, anti-TIAR antibodies, and protein G sepharose beads (Cell Signaling) according to the manufacturers’ protocols and our previous study [22]. Cellular RNA was pulled down with the antibodies and pre-immune rabbit IgG (supplied with the kit). The co-precipitated RNA was purified and sequentially subjected to RT-qPCR to detect TPD52 and β-actin.

### Cell growth and apoptosis assay

Five hundred cells were seeded on a 96-well tissue culture plate and cultured for 24 h under normoxia. The cells were then incubated under hypoxic conditions. After 1, 2, and 3 d, the cells were subjected to tetrazolium salt (3-(4,5-Dimethylthiazol-2-yl)-2,5-diphenyltetrazolium bromide) (MTT) and caspase 3/7 assays, as described previously [19].

### Stable clones

SAS cells were infected with MISSION shRNA lentivirus TurboGFP shRNA for TPD52 (TRCN0000158761, Sigma-Aldrich) and MISSION control TurboGFP lentivirus (SHC003V, Sigma-Aldrich). The infected cells were selected using 1 mg/mL puromycin (Sigma-Aldrich) and each single clone was isolated according to the manufacturer’ s protocols. Expression of TurboGFP and knockdown of TPD52 were confirmed by fluorescence microscopy and RT-qPCR, respectively. The cells were then maintained in HDMEM supplemented with 10% FBS and 1 mg/mL puromycin.

### Mice

This study was approved by the Animal Care and Use Committee (Approval No. 12009) and was conducted according to the Showa University Animal Guidelines for Animal Experiments. Four-week-old female BALB/c *nu/nu* mice were purchased from Claire Japan (Tokyo, Japan) and maintained under pathogen-free conditions. Approximately 10^6^ cells in 100 µL saline were subcutaneously injected into a unilateral flank, and the cancer-bearing mice were maintained for 7 d in order to grow tumors. Thereafter, mice were divided into four groups (i.e., Control, Control-PX478, shTPD52, and shTPD52-PX-478, n = 3 in each experimental group), the day was designated as “day 0.” Every three days, tumor volumes and body weights were measured, and saline (100 ml) or 0.5 mg/kg body weight of PX-478 (Cayman, Ann Arbor, MI) in 100 ml saline was intraperitoneally injected. On day 18, mice were euthanized using CO_2_ asphyxiation, and the tumors were removed for histochemistry. Tumor volume was determined by direct measurement and calculated using the formula π/6 × (large diameter) × (small diameter)^2^ [19].

### Immunohistochemistry

Resected specimens were fixed with 10% formalin, embedded in paraffin, stained with hematoxylin and eosin (H-E) (Sakura Finetek Japan, Tokyo, Japan), and then immunohistochemically stained for p62, Akt, and phospho-Akt, as described previously [19]. Details regarding clinical samples and immunofluorescence experiments can be found in Additional file 1.

### Statistics

Unless otherwise specified, all experiments were repeated at least three times, and similar results were obtained in the repeated experiments. Statistical analyses were performed by analysis of variance (ANOVA) methods. Significance was determined at *, *p* < 0.05. All analyses were performed with a commercial computer software, KaleidaGraph version 4.5 (Hulinks, Tokyo, Japan).

## Supplementary Information

**Additional file 1.** Supplementary methods.

**Additional file 2: Table S1.** Primer pairs (sense and antisense strands) used for RT-qPCR in the present study.

**Additional file 3: Fig S1.** Expression of HIF-1α, TPD52, −53, and −54 in a representative oral squamous carcinoma specimen. Representative resected squamous carcinoma tissues from the tongue were stained with H-E, and were immunohistochemically stained for HIF-1α, TPD52, −53, −54. Optical microscopic images were captured at low (× 100) and high (× 200) magnification. Bars, 200 μm. **Fig S2.** Effects of hypoxia on the formation of stress granules. SAS cells were seeded on tissue culture chamber slides and exposed to hypoxia for 24 h. Then, the cells were subjected to DAPI staining and immunofluorescence for TIA-1 and TIAR. Merged images are shown on the right side. Bar, 50 mm.

## Data Availability

The data used to support the findings of the present study are available from the corresponding author upon request.
