# Supplementary material for: Tumor protein D52 is upregulated in oral squamous carcinoma cells under hypoxia in a hypoxia-inducible-factor-independent manner and is involved in cell death resistance
Source: Cell Biosci. 2021 Jul 3;11:122. doi: 10.1186/s13578-021-00634-0 (PMC8255020; doi:10.1186/s13578-021-00634-0)
Supplement: Supplementary file 1 — Additional file 1. Supplementary methods. [file 13578_2021_634_MOESM1_ESM.docx]

**Tumor protein D52 is upregulated in oral squamous carcinoma cells under hypoxia in a hypoxia-inducible-factor-independent manner and is involved in cell death resistance**

Yuzo Abe, Yoshiki Mukudai, Mai Kurihara, Asami Houri, Junichiro Chikuda, Atsutoshi Yaso, Kosuke Kato, Toshikazu Shimane, Tatsuo Shirota

Department of Oral and Maxillofacial Surgery, School of Dentistry, Showa University, 2-1-1 Kitasenzoku, Ota-ku, Tokyo 145-8515, Japan

Corresponding author: Yoshiki Mukudai

E-mail: [mukudai@dent.showa-u.ac.jp](mailto:mukudai@dent.showa-u.ac.jp)

**Supplementary Methods**

**Clinical samples**

All samples were acquired from patients who underwent treatment for squamous cell carcinoma in Showa University Dental Hospital from January 2001 through March 2015. All patients provided informed consent before enrollment in the study, in accordance with the protocol approved by the Institutional Review Board at Showa University Dental Hospital (approval no. DH2015-013). Primary lesions were resected from tongue. The specimens were stained with H-E, and then, immunohistochemically stained for HIF-1α, TPD52, 53 and 54, as described in another subsection.

**Immunofluorescence (IF)**

The cells were seeded in a collagen-coated-slide chamber (IWAKI) at a density of 3 ×103 cells/well, then incubated and exposed to hypoxia, as described in another subsection. For IF, the cells were rinsed with phosphate-buffered saline (PBS) three times, fixed with 4% paraformaldehyde (Wako) for 10 min at room temperature, permeabilized with 0.5% TritonX-100 (Sigma Aldrich) for 10 min at room temperature, and washed another three times. Anti-TIA-1 and anti-TIAR antibodies in PBS were added to each well and samples were incubated at room temperature overnight. After washing with PBS three times, fluorescein-conjugated secondary antibody (Alexa Flour 488 goat anti-rabbit IgG, Thermo Fisher) was added and incubated in the dark for 1 h. Cell nuclei were stained with 4’.6- diamidino-2-phenylindole (DAPI) and visualized using a fluorescence microscope (BZ-9000, KEYENCE, Osaka, Japan).
