# Supplementary material for: Tumor protein D52 is upregulated in oral squamous carcinoma cells under hypoxia in a hypoxia-inducible-factor-independent manner and is involved in cell death resistance
Source: Cell Biosci. 2021 Jul 3;11:122. doi: 10.1186/s13578-021-00634-0 (PMC8255020; doi:10.1186/s13578-021-00634-0)
Supplement: Supplementary file 3 — Additional file 3: Fig S1. Expression of HIF-1α, TPD52, −53, and −54 in a representative oral squamous carcinoma specimen. Representative resected squamous carcinoma tissues from the tongue were stained with H-E, and were immunohistochemically stained for HIF-1α, TPD52, −53, −54. Optical microscopic images were captured at low (× 100) and high (× 200) magnification. Bars, 200 μm. Fig S2. Effects of hypoxia on the formation of stress granules. SAS cells were seeded on tissue culture chamber slides and exposed to hypoxia for 24 h. Then, the cells were subjected to DAPI staining and immunofluorescence for TIA-1 and TIAR. Merged images are shown on the right side. Bar, 50 mm. [file 13578_2021_634_MOESM3_ESM.docx]

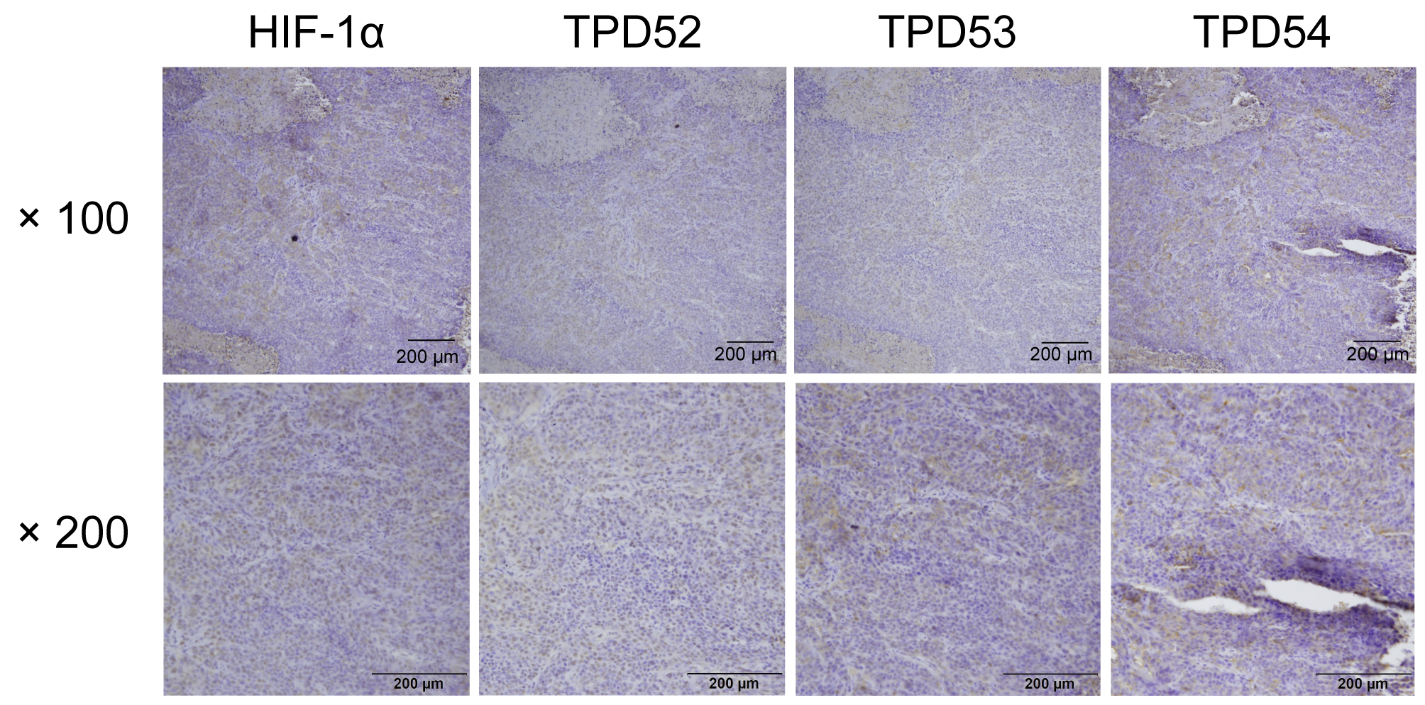


Fig S1**.** Expression of HIF-1α, TPD52, -53, and -54 in a representative oral squamous carcinoma specimen. Representative resected squamous carcinoma tissues from the tongue were stained with H-E, and were immunohistochemically stained for HIF-1α, TPD52, -53, -54. Optical microscopic images were captured at low (x 100) and high (x 200) magnification. Bars, 200 μm.


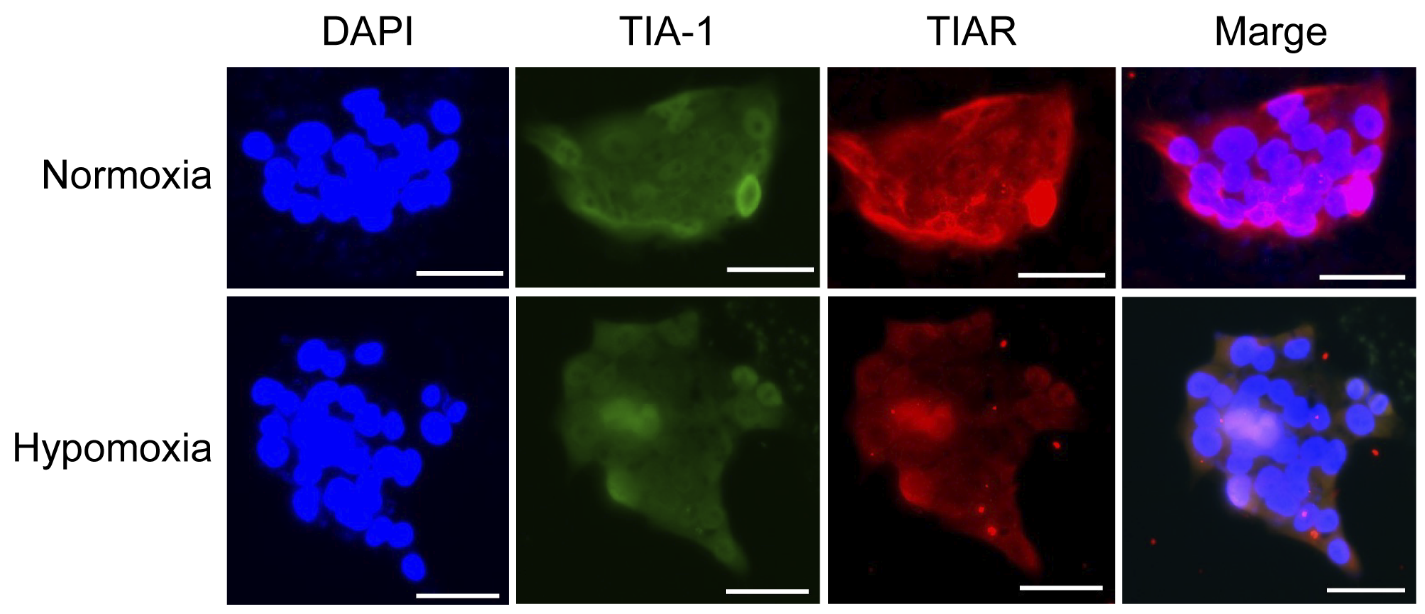


Fig S2. Effects of hypoxia on the formation of stress granules. SAS cells were seeded on tissue culture chamber slides and exposed to hypoxia for 24 h. Then, the cells were subjected to DAPI staining and immunofluorescence for TIA-1 and TIAR. Merged images are shown on the right side. Bar, 50 mm.
